# Synchronicity of viral shedding in molossid bat maternity colonies

**DOI:** 10.1017/S0950268823000171

**Published:** 2023-02-08

**Authors:** Axel O. G. Hoarau, Marie Köster, Muriel Dietrich, Gildas Le Minter, Léa Joffrin, Riana V. Ramanantsalama, Patrick Mavingui, Camille Lebarbenchon

**Affiliations:** Université de La Réunion, Processus Infectieux en Milieu Insulaire Tropical, Inserm 1187, CNRS 9192, IRD 249, Sainte-Clotilde, La Réunion, France

**Keywords:** Astrovirus, coronavirus, *Mormopterus francoismoutoui*, paramyxovirus, Reunion Island

## Abstract

Infection dynamics in vertebrates are driven by biological and ecological processes. For bats, population structure and reproductive cycles have major effects on RNA virus transmission. On Reunion Island, previous studies have shown that parturition of pregnant females and aggregation of juvenile Reunion free-tailed bats (*Mormopterus francoismoutoui*) are associated with major increase in the prevalence of bats shedding RNA viruses. The synchronicity of such shedding pulses, however, is yet to be assessed between viruses but also maternity colonies. Based on 3422 fresh faeces collected every 2–5 weeks during four consecutive birthing seasons, we report the prevalence of bats shedding astroviruses (AstVs), coronaviruses (CoVs) and paramyxoviruses (PMVs) in two maternity colonies on Reunion Island. We found that the proportion of bats shedding viruses is highly influenced by sampling collection periods, and therefore by the evolution of the population age structure. We highlight that virus shedding patterns are consistent among years and colonies for CoVs and to a lesser extent for PMVs, but not for AstVs. We also report that 1% of bats harbour co-infections, with two but not three of the viruses, and most co-infections were due to CoVs and PMVs.

## Introduction

Seasonal infection dynamics in vertebrates are driven by a large set of biological and ecological factors, including social behaviours and changes in host population structure [1]. For instance, the breeding season sometimes leads to the aggregation of hundreds to thousands of bats in maternity colonies, generating highly conducive conditions for the transmission of infectious agents [2–8]. Identifying the factors involved in the transmission dynamics of bat-borne pathogens expands our understanding of host–parasite interactions, but is also critical to assess spill-over potential to other hosts [9, 10].

Although many studies have reported viruses, bacteria and blood parasites in bats [11–13], a precise assessment of the temporal variation in their circulation in host populations remains challenging [14]. In addition to host-related factors, those associated to the bat colony (e.g. type of habitat, occupation length) and to the simultaneous circulation of multiple infectious agents have not been fully explored. Although co-infections (i.e. presence of at least two infectious agents in the same host) are known to affect host susceptibility or ability to maintain and transmit infectious agents [15–21], their role in infectious agent transmission dynamics in bat populations indeed remains unsolved.

In this study, we investigated the effect of population size, occupation length and age structure on virus shedding, in molossid bat maternity colonies. Because reproductive cycles are highly synchronised, we hypothesised that viral shedding patterns could be similar and predictable between years. However, differences may be expected between viral families, and associated to either epidemiological or ecological factors such as herd immunity or the maintenance of viral particles in the environment. We also assumed that viral co-circulation in bat populations may generate positive or negative interactions between viruses, with cascade effects on their transmission dynamics.

To test these hypotheses, we focused on the Reunion free-tailed bat (RFTB; *Mormopterus francoismoutoui*), a molossid species endemic to Reunion Island. We collected 3422 fresh faeces simultaneously in two maternity colonies, during four consecutive years, and estimate the prevalence of astroviruses (AstVs), coronaviruses (CoVs) and paramyxoviruses (PMVs). More precisely, (i) we assessed temporal variations in the prevalence of bats shedding viruses, (ii) estimated the proportion of co-infected bats and (iii) tested whether the detection of a given viral family was associated to the presence of another.

## Methods

### Study sites

The study was conducted in two monospecific RFTB colonies on Reunion Island, a 2500 km^2^ oceanic island located in the South-Western Indian Ocean. The first colony was located in a ~30 m^3^ natural cave on the West coast of the island (referred to hereafter as ‘cave’). This colony contains the highest number of bats on the island, and is occupied only during the birthing season, from October to June [8]. During the early stages of the season (October to December), the colony is mainly composed of pregnant females, with a population reaching about 40 000–50 000 flying bats before parturition (starting in mid-December) [8]. These females gradually leave the colony approximately 1 month after parturition. Then, from January to May, the colony is mainly composed of newborns and juveniles. Once all juveniles have left the cave, it remains empty until the following maternity season. As for previous studies [8, 22], population age structure and parturition timing was visually monitored based on bat morphology. Fur coloration was shown to be a reliable indicator during the early stage of the season (adults: brown fur; newborns: nude pink skin; juveniles: dark grey fur).

A smaller colony (up to 1200 flying bats) was also monitored, in a ~5 m^3^ building housing a power transformer, located in the North coast of the island (referred to hereafter as ‘building’). In this colony, adults are reported all year long but newborns are present in mid-December, and juveniles between January and May, as for the cave colony. The building remains occupied between June and September corresponding to the austral winter, although it is not possible to visually assess the population age structure at this stage of the season. Juveniles’ fur coloration starts to change and differences between juveniles and adults cannot be discriminated based on fur coloration only.

The cave colony was monitored during four consecutive years (i.e. each year corresponding to a birthing season), from October 2016 to March 2020. The building colony was monitored during three consecutive years, from November 2017 to October 2020. Because of the lockdown associated with the COVID19 pandemic, sampling was not done in March and April 2020.

### Sample collection

Fresh bat faecal pellets were collected every 2–5 weeks, and during the same day in both colonies (except for nine sampling sessions, because of bad weather limiting bat emergence from the building). For the cave colony, samples were collected in the morning, by placing Benchguard® sheets (60 cm × 49 cm) (Thermo Fisher Scientific, Waltham, MA, USA) under roosting bats [22]. For the building colony, samples were collected during bat emergence, at dusk, due to access restriction to the power transformer. Plastic trays with Benchguard® strips (12 cm × 35 cm) were placed below the colony exit. For both colonies, faeces were individually placed into a tube containing 1.5 ml of brain heart infusion medium (Conda, Madrid, Spain) supplemented with penicillin G (1000 U/ml), streptomycin (1 mg/ml), kanamycin (0.5 mg/ml), gentamicin (0.25 mg/ml) and amphotericin B (0.025 mg/ml). Samples were maintained refrigerated in the field and then stored at –80°C in the laboratory.

In total, 3422 fresh faeces were collected from the two colonies during 64 sampling sessions (Supplementary Table S1). In summary, 580 samples were collected in the cave between October 2016 and June 2017 for the first season, during 14 sampling sessions. For the second season, a total of 964 faeces were collected in both sites (cave: *n* = 499; building: *n* = 465) between November 2017 and October 2018, during 17 sampling sessions. Nine hundred and eighty faeces were collected (cave: *n* = 525; building: *n* = 455) between November 2018 and September 2019 during 17 sampling sessions. Finally, 898 samples were collected (cave: *n* = 440; building: *n* = 458) between October 2019 and October 2020 during 16 sampling sessions.

### RNA extraction and cDNA synthesis

Samples were thawed at 4°C overnight, briefly vortexed and centrifuged at 1500 *g* for 15 min. RNA extraction was performed with the IndiSpin QIAcube HT Pathogen Kit as recommended (QIAGEN, Hilden, Germany). Reverse-transcription was performed on 10 μl of RNA, with the ProtoScript II Reverse Transcriptase and Random primers 6 (New England BioLAbs, Ipswich, MA, USA), as previously described [23].

### Virus detection

cDNAs were tested for the presence of the AstVs RNA-dependent RNA-polymerase (RdRp) gene using a Pan-AstV semi-nested polymerase chain reaction (PCR), following a previously published protocol [24] routinely used in our laboratory [23, 25, 26]. PCRs were performed with the GoTaq G2 Hot Start Green Master Mix (Promega, Madison, WI, USA) in an Applied Biosystems 2720 Thermal Cycler (Thermo Fisher Scientific). Electrophoresis was performed on 2% agarose gels stained with 2% Gelred (Biotium, Hayward, CA, USA). All PCR products of the expected size were submitted for direct Sanger sequencing (Genoscreen, Lille, France) in order to confirm sample positivity.

cDNAs were tested for the presence of the CoVs RdRp gene using a Pan-CoV multi-probe real-time (RT) PCR following a previously published protocol [27] routinely used in our laboratory [22, 28]. PCRs were performed with the QuantiNova Probe PCR Master Mix (QIAGEN) in a CFX96 Touch™ Real-Time PCR Detection System (Bio-Rad, Hercules, CA, USA). Samples collected in the cave between October 2016 and June 2018 were tested for CoV RNA as part of a previous study [22]. A subset of positive samples was submitted for direct Sanger sequencing in 2016–2017 and in 2017–2018 [22] . All of the RT-PCR-positive samples that were sequenced were confirmed to be positive; we therefore did not sequence further RT-PCR amplicons.

cDNAs were tested for the presence of the PMVs L-polymerase gene targeting *Respiroviruses*, *Morbilliviruses* and *Henipaviruses* (RMH) using a semi-nested PCR following a previously published protocol [29] routinely used in our laboratory [8, 30, 31], but with slight modifications. A 10-fold dilution of amplicons obtained in the first PCR was used to perform the second PCR and increase specificity. PCRs were performed with the GoTaq G2 Hot Start Green Master Mix (Promega) in an Applied Biosystems 2720 Thermal Cycler (Thermo Fisher Scientific). Electrophoresis was performed on agarose as above, and all PCR products of the expected size were also submitted for direct Sanger sequencing to confirm sample positivity.

### Statistical analysis

Analyses were made with the assumption that each faeces came from an individual bat. Based on the high number of bats (hundreds in the buildings to tens of thousands in the cave), together with the high bat density (>900 individuals per m^2^) [32], and the limited time spent in the colony for sampling (<15 min), we considered that the probability to collect two or more faeces from the same bat was limited, although this could not be formally excluded. Our previous study on CoV shedding dynamics in the cave colony using a similar sampling scheme [22] provided consistent results, supporting the efficacy of such non-invasive methodology to assess the prevalence of bat shedding viruses.

Pearson *χ*^2^ tests were conducted to globally examine the effect of the colony and the year of collection on virus detection. Then, four generalised linear mixed models (GLMMs) with binomial error structures were used to explore the effect of the colony (different population sizes and occupation lengths) and the sampling collection period, respectively, on the detection of AstVs, CoVs and PMVs RNA, as well as co-infections, with the year (i.e. season of parturition) included as a random factor. Five sampling collection periods were defined based on the biology of the studied species, and the evolution of the population age structure. Period A ranged from the beginning of the birthing season to the parturition. Period B corresponded to the birth period. Period C represented weeks during which newborns become juveniles. Period D corresponded to the departure of adults after juveniles’ weaning. Finally, period E represented the austral winter, when only the building remains occupied. Analyses were conducted only when data were available for both sites (i.e. period A to period D for 2017–2018, 2018–2019 and 2019–2020). Three generalised linear models with binomial error and a logit link function were used to test if the presence/absence of each virus was influenced by those of the other viruses. Analyses were conducted in R version 4.1.0 [33] with the package ‘*lme4*’ [34]. The significance of each variable was determined with the ‘*Anova*’ function from the ‘*car*’ package [35].

## Results

### Bat population age structure in the colonies

Adult bats were observed between October and December, in both the cave and the building colonies. Newborns were sighted in both colonies from mid-December to late January, suggesting that parturition was highly synchronised between the two maternity colonies (each year, newborns were sighted for the first time, during the same sampling session in both the cave and the building). From February to May, both colonies were mainly composed of weaned juvenile bats, following the departure of adults approximately 1 month after parturition. No bats were observed in the cave between mid-June and September (austral winter). These observations are consistent with previous studies [8, 22].

### Infection dynamics

**Fig. 1.**
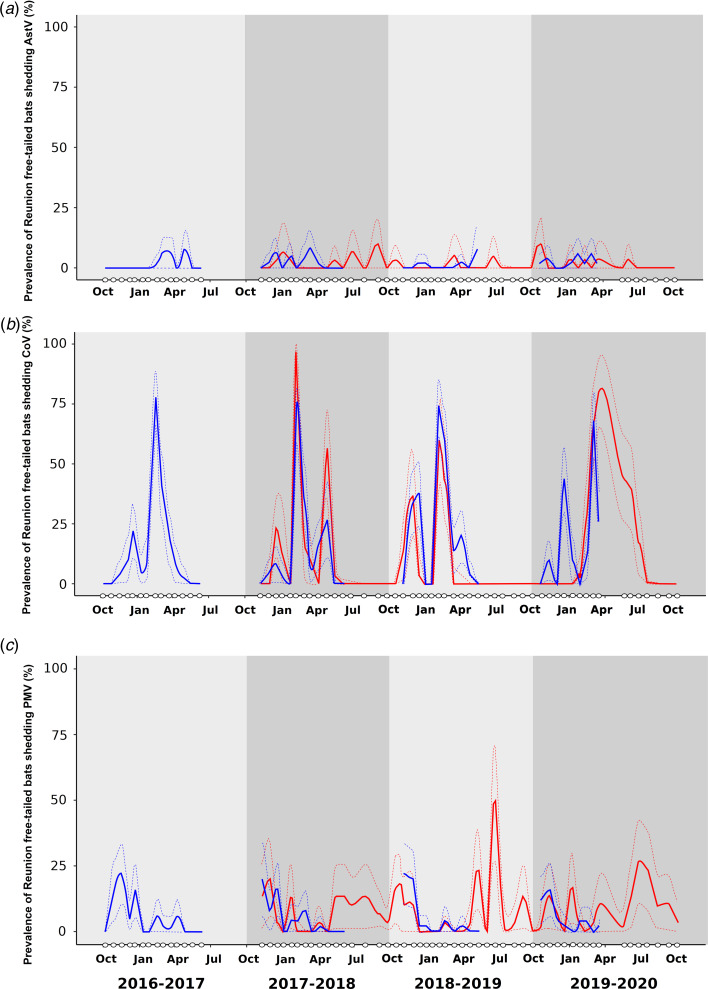
Prevalence of Reunion free-tailed bat (*Mormopterus francoismoutoui*) shedding astrovirus (AstV; a), coronavirus (CoV; b) and paramyxovirus (PMV; c), during four consecutive seasons (October 2016 to October 2020). White dots on *x*-axis indicate sampling dates. Continuous lines represent the proportion of positive samples and were predicted with a loess function in R. Dashed lines represent the 95% confidence interval. Blue: cave (occupied by bats from October to May); red: building (occupied all year long).

AstVs were detected in both colonies, every year (Supplementary Table S1, Fig. 1a). In total, 57 samples tested positive (mean detection rate ± 95% confidence interval: 1.7 ± 0.4%). Globally, a slight variation in AstV RNA detection was found between years (*χ*^2^ = 11.79, df = 3, *P* < 0.01). The prevalence of bats shedding AstVs was lower in 2019–2020. However, no significant difference was observed between colonies (*χ*^2^ = 1.19, df = 1, *P* = 0.28). The GLMM did not reveal any significant variation between sampling collection periods (*χ*^2^ = 2.49, *P* = 0.48), nor colonies (*χ*^2^ = 1.26, *P* = 0.26).

CoVs were detected in both colonies, every year (Supplementary Table S1, Fig. 1b). In total, 573 samples tested positive (16.7 ± 1.3%). Globally, a significant variation in CoV RNA detection was found between the two sites (*χ*^2^ = 14.74, df = 1, *P* < 0.001). The global prevalence was lower in the building (13.8 ± 1.9%) as compared to the cave (18.7 ± 1.7%). However, no significant difference was observed between years (*χ*^2^ = 5.29, df = 3, *P* = 0.15). The GLMM highlighted major variations in the prevalence of bats shedding CoVs between the sampling collection periods (*χ*^2^ = 255.56, *P* < 0.001), however no significant variations was detected between colonies (*χ*^2^ = 0.02, *P* = 0.88). The proportion of bats shedding CoV RNA was significantly higher for the periods C (50.5 ± 5.0%), D (20.1 ± 2.9%) and A (12.2 ± 2.3%). For period A, a shedding pulse was recorded in December almost all years for both colonies (e.g. ranging from 8.0 ± 7.5% to 38.0 ± 13.5% in the cave), except in 2019–2020 in the building for which no samples tested positive during the period (Fig. 1b). For period C, another shedding pulse was recorded in February (e.g. ranging from 72.0 ± 12.5% to 78.0 ± 11.5% in the cave). This second pulse was delayed in March (period D), for both colonies, in 2019–2020 (respectively 68.0 ± 13.0% and 80.0 ± 14.3% of bats shedding CoVs in the cave and in the building) (Supplementary Table S1). None of the samples tested positive during the austral winter (period E).

PMVs were detected in both colonies, every year (Supplementary Table S1, Fig. 1c). In total, 224 samples (6.5 ± 0.8%) tested positive. Globally, a significant variation in PMV RNA detection was found between the two sites (*χ*^2^ = 11.03, df  = 1, *P* < 0.001). The global prevalence was lower in the cave (5.4 ± 1.0%) as compared to the building (8.3 ± 1.5%). However, no significant difference was observed between years (*χ*^2^ = 2.19, df = 3, *P* = 0.53). The GLMM revealed major variations in the prevalence of bats shedding PMVs between the sampling collection periods (*χ*^2^ = 35.41, *P* < 0.001), however no significant variations were detected between colonies (*χ*^2^ = 1.31, *P* = 0.25). The proportion of bats shedding PMV RNA was significantly higher for the period A (10.7 ± 2.2%). A strong interaction between the sampling collection period and the colony was also identified (*χ*^2^ = 18.28, *P* < 0.001). Interestingly, PMV RNA was also detected during the austral winter (period E) in the building (e.g. up to 50.0 ± 21.9% in June in 2018–2019) (Fig. 1c).

### Co-infections

**Fig. 2.**
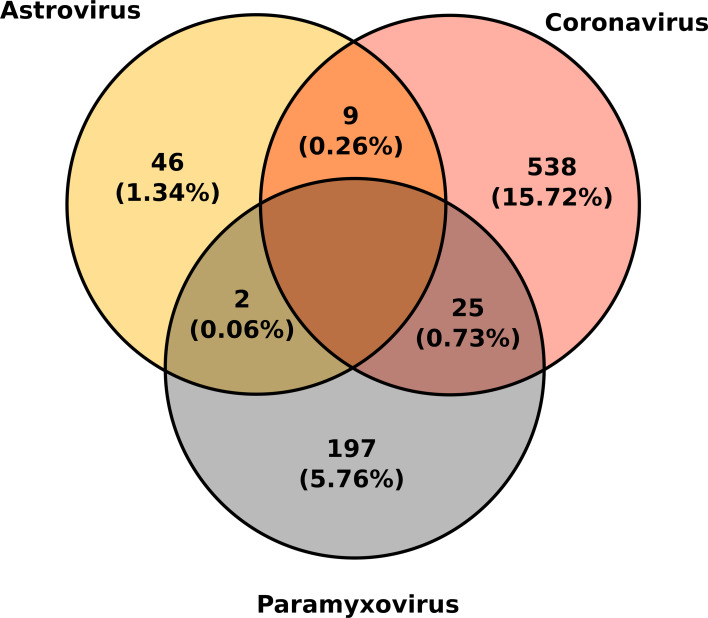
Co-infections between astroviruses, coronaviruses and paramyxoviruses in Reunion free-tailed bat (*Mormopterus francoismoutoui*). The number and proportion of positive samples is indicated for each virus.

Co-infections were detected in both colonies and every year. In total, 36 of the 3422 samples tested positive for more than one virus (1.1 ± 0.3%; Fig. 2). Globally, no significant variation in co-infection detection was found between colonies (*χ*^2^ = 0.26, df = 1, *P* = 0.61), nor between years (*χ*^2^ = 4.98, df = 3, *P* = 0.17). Only bi-infections were observed. Co-infections with CoVs and PMVs (0.7 ± 0.3%) were more common than those with AstVs and PMVs (0.1 ± 0.1%) or AstVs and CoVs (0.3 ± 0.2%) (*χ*^2^ = 22.19, df = 2, *P* < 0.001). GLMM did not reveal any significant variation in the prevalence of bats shedding co-infections between sampling collection periods (*χ*^2^ = 0.73, *P* = 0.86), nor between colonies (*χ*^2^ = 0.04, *P* = 0.83). Additionally, there was no statistical association between viruses indicating that the presence of one virus may not be determined by the presence of another (Supplementary Table S2).

## Discussion

Based on the molecular detection of AstVs, CoVs and PMVs, we precisely assessed temporal variations in virus shedding dynamics in two RFTB colonies during four consecutive years. In spite of differences between colonies, notably population size (up to 50 000 *vs.* 1200 flying bats), and occupation length (maternity period *vs.* permanent colony), a similar shedding pattern was observed between colonies for CoVs and, to a lesser extent, for PMVs. Strong variations were observed among sampling collection periods and could be related to the evolution of the population age structure and the presence of naive individuals throughout the birthing season, or to broader environmental and/or biological factors affecting herd immunity. We also detected a slight proportion of co-infected bats, with CoVs and PMVs co-infections more commonly detected than AstVs and CoVs, or AstVs and PMVs.

A major shedding pulse was consistently observed a few weeks after parturition in both colonies for CoVs. It was detected in February for the first 3 years of the follow-up, and in March for the last year. The similarities among colonies suggest that population size may not affect CoVs transmission in RFTB. However, age structure and the presence of naive individuals could be one of the drivers of the epidemiological process [22]. This major shedding pulse, occurring when the colonies were mainly composed of juveniles, could be associated with the waning of maternal antibodies as previously suggested for a colony of Greater Mouse-eared bats (*Myotis myotis*) in Germany [5]. The transmission of maternal immunity seems to be a common phenomenon in bat populations [36, 37], but to date, the transmission and persistence of CoV maternal antibodies in molossid bats remain to be assessed [38]. Interestingly, in 2019–2020, this major shedding pulse was slightly delayed in both colonies and was detected in March, not February. This result could reflect a subtle modification in the reproduction cycle such as a late arrival of pregnant females driven by bad environmental conditions or delayed mating, resulting in delayed births and waning of maternal antibodies in juvenile bats. This hypothesis could not be verified with our sampling approach but further longitudinal studies including captures and investigating RFTB population dynamics at the island scale may help to clarify these assumptions. Shedding pulses were also detected earlier in the season in period A, at the time of parturition, when the colonies were mainly composed of pregnant females. These pulses may reflect a transient pregnancy-related depressed immunity, as suggested in other studies [5, 8, 22]. This pulse was also repeatable between years and synchronised between colonies, although not detected in the building in 2019–2020. This might also reflect a subtle variation in the reproduction and could also correspond to a late arrival of pregnant females in the colony, corroborating the slightly delayed shedding pulse detected in juvenile bats, later in the season.

Repeated shedding pulses were also detected for PMVs, although with a lower amplitude than for CoVs. Synchronised increases were observed between colonies, at the beginning of the birthing season in November and could be associated to the presence of immune depressed pregnant females as for CoVs, and as previously already suggested in another study investigating PMV infection dynamics in the cave [8]. Although more inconsistently, PMVs were also detected after parturition, between January and May. These increases were minor as compared to CoVs, but may also be associated to the waning of maternal antibodies in juvenile bats [8]. The discrepancies observed between CoVs and PMVs could result from different immune responses towards the given infectious agents, but could also result from differences in transmission rates [22]. Additionally, a statistical interaction between colony and sampling collection periods was also detected for PMVs, supporting that differences in the host population structure may exist throughout the birthing season among the two sites and leading to different levels of PMV transmission.

In addition to the variations in the prevalence of bats shedding PMVs and CoVs, we also highlighted a major difference among the two viruses after the birthing season, between June and October. Indeed, PMVs were detected in the building, every year, during the austral winter, but not CoVs. This discrepancy raises questions about their maintenance in bat populations. PMVs may be maintained in bat populations by circulating in roosting habitats mostly composed by juvenile bats during the austral winter, allowing immune-depressed pregnant females to be infected at the beginning of the following birthing season. For CoVs, the maintenance could be driven by an environmental persistence of viral particles during the austral winter within the colonies, leading to yearly exposure of susceptible individuals at the beginning of the birthing season. This assumption could be supported by the detection of CoV RNA in dry guano samples up to 3 months after the bats’ departure in the cave in our previous study [22]. However, studies focusing on bat population structure as well as on the isolation of infectious viral particles from the environment are needed to fully assess these mechanisms. Mathematical modeling approaches could also be considered to investigate the transmission dynamics of these viruses.

For AstVs, no significant variation was observed in the detection of RNA throughout the birthing season. Additionally, the global prevalence was lower than for CoVs and PMVs. These results contrast with a previous longitudinal study conducted in Germany, in a Greater Mouse-eared bat (*Myotis myotis*) maternity colony, reporting a higher prevalence and AstV shedding pulses early in the birthing season [5]. These differences support that host susceptibility and immunological responses depend on bat species and viruses, even among a same viral family [39].

Of the few co-infections detected, most involved CoVs and PMVs, and may be explained by the higher proportions of bats shedding these two viruses, as compared to AstVs. We did not report any interaction among viruses. In Borneo, a study conducted in eight tropical species reported co-infections and interactions between AstVs and CoVs in Fawn Leaf-nosed bats (*Hipposideros cervinus*) [16]. We focused on the co-circulation of three viral families, but bats are natural hosts of a large diversity of infectious agents, potentially interacting [11, 40]. The RFTB has, for example, been found to host *Leptospira* sp. bacteria whose presence is positively associated with the presence of PMVs [8]. Experimental approaches such as next generation sequencing, or metagenomics, may provide the opportunity to better characterise the range of infectious agents that may be involved in co-infections [18, 19], and their consequences on transmission dynamics.

Overall, this study depicts a strong seasonality of the infection dynamics in a tropical bat population for CoVs and PMVs, but not for AstVs. Different shedding patterns between viral families were found, with predictable infection patterns among years for CoVs. We suggest that these patterns could be driven by the evolution of the population age structure and to the presence of immunologically naive individuals. These findings further underline the need to assess the drivers involved in virus transmission dynamics in bats, beyond host-related factors, by focusing on the interactions with the environment and other infectious agents. Such a global assessment is needed to explore spill-over risk associated with major shedding pulses in bat populations [9, 19].

## Data Availability

Data are available in the Supplementary material.

## Supplementary material

For supplementary material accompanying this paper visit http://doi.org/10.1017/S0950268823000171. click here to view supplementary material

## References

[1] Altizer S (2006) Seasonality and the dynamics of infectious diseases. Ecology Letters 9, 467–484. 16623732 10.1111/j.1461-0248.2005.00879.x

[2] Peel AJ (2014) The effect of seasonal birth pulses on pathogen persistence in wild mammal populations. Proceedings of the Royal Society B: Biological Sciences 281, 20132962. 10.1098/rspb.2013.2962 PMC4046395 24827436

[3] Lloyd-Smith JO (2005) Should we expect population thresholds for wildlife disease? Trends in Ecology & Evolution 20, 511–519. 16701428 10.1016/j.tree.2005.07.004

[4] Plowright RK (2008) Reproduction and nutritional stress are risk factors for Hendra virus infection in little red flying foxes (*Pteropus scapulatus*). Proceedings of the Royal Society B: Biological Sciences 275, 861–869. 10.1098/rspb.2007.1260 PMC2596896 18198149

[5] Drexler JF (2011) Amplification of emerging viruses in a bat colony. Emerging Infectious Diseases 17, 449–456. 21392436 10.3201/eid1703.100526 PMC3165994

[6] Langwig KE (2012) Sociality, density-dependence and microclimates determine the persistence of populations suffering from a novel fungal disease, white-nose syndrome. Ecology Letters 15, 1050–1057. 22747672 10.1111/j.1461-0248.2012.01829.x

[7] Hayman DTS (2015) Biannual birth pulses allow filoviruses to persist in bat populations. Proceedings of the Royal Society B: Biological Sciences 282, 20142591. 10.1098/rspb.2014.2591 PMC4345444 25673678

[8] Dietrich M (2015) *Leptospira* and paramyxovirus infection dynamics in a bat maternity enlightens pathogen maintenance in wildlife: dual infection dynamics in a bat maternity. Environmental Microbiology 17, 4280–4289. 25580582 10.1111/1462-2920.12766

[9] Amman BR (2012) Seasonal pulses of Marburg virus circulation in juvenile *Rousettus aegyptiacus* bats coincide with periods of increased risk of human infection. PLoS Pathogens 8, e1002877. 23055920 10.1371/journal.ppat.1002877 PMC3464226

[10] Brook CE and Dobson AP (2015) Bats as ‘special’ reservoirs for emerging zoonotic pathogens. Trends in Microbiology 23, 172–180. 25572882 10.1016/j.tim.2014.12.004 PMC7126622

[11] Calisher CH (2006) Bats: important reservoir hosts of emerging viruses. Clinical Microbiology Reviews 19, 531–545. 16847084 10.1128/CMR.00017-06 PMC1539106

[12] Schaer J (2013) High diversity of West African bat malaria parasites and a tight link with rodent *Plasmodium* taxa. Proceedings of the National Academy of Sciences of the USA 110, 17415–17419. 24101466 10.1073/pnas.1311016110 PMC3808598

[13] Veikkolainen V (2014) Bats as reservoir hosts of human bacterial pathogen, *Bartonella mayotimonensis*. Emerging Infectious Diseases 20, 960–967. 24856523 10.3201/eid2006.130956 PMC4036794

[14] Mortlock M (2021) Seasonal shedding patterns of diverse henipavirus-related paramyxoviruses in Egyptian rousette bats. Scientific Reports 11, 24262. 34930962 10.1038/s41598-021-03641-w PMC8688450

[15] Davy CM (2018) White-nose syndrome is associated with increased replication of a naturally persisting coronaviruses in bats. Scientific Reports 8, 15508. 30341341 10.1038/s41598-018-33975-x PMC6195612

[16] Seltmann A (2017) Seasonal fluctuations of astrovirus, but not coronavirus shedding in bats inhabiting human-modified tropical forests. EcoHealth 14, 272–284. 28500421 10.1007/s10393-017-1245-x PMC7087689

[17] Cox FEG (2001) Concomitant infections, parasites and immune responses. Parasitology 122, S23–S38. 11442193 10.1017/s003118200001698x

[18] Vaumourin E (2015) The importance of multiparasitism: examining the consequences of co-infections for human and animal health. Parasites & Vectors 8, 545. 26482351 10.1186/s13071-015-1167-9 PMC4617890

[19] Hoarau AOG, Mavingui P and Lebarbenchon C (2020) Coinfections in wildlife: focus on a neglected aspect of infectious disease epidemiology. PLoS Pathogens 16, e1008790. 32881983 10.1371/journal.ppat.1008790 PMC7470396

[20] Petney TN and Andrews RH (1998) Multiparasite communities in animals and humans: frequency, structure and pathogenic significance. International Journal for Parasitology 28, 377–393. 9559357 10.1016/s0020-7519(97)00189-6

[21] Jolles AE (2008) Interactions between macroparasites and microparasites drive infection patterns in free-ranging African buffalo. Ecology 89, 2239–2250. 18724734 10.1890/07-0995.1

[22] Joffrin L (2022) Seasonality of coronavirus shedding in tropical bats. Royal Society Open Science 9, 211600. 35154796 10.1098/rsos.211600 PMC8825989

[23] Lebarbenchon C (2017) Astroviruses in bats, Madagascar. Emerging Microbes & Infections 6, e58. 28634357 10.1038/emi.2017.47 PMC5520320

[24] Chu DKW (2008) Novel astroviruses in insectivorous bats. Journal of Virology 82, 9107–9114. 18550669 10.1128/JVI.00857-08 PMC2546893

[25] Hoarau F (2018) Bat astrovirus in Mozambique. Virology Journal 15, 104. 29925396 10.1186/s12985-018-1011-x PMC6011250

[26] Joffrin L (2021) Astrovirus in Reunion free-tailed bat (*Mormopterus francoismoutoui*). Viruses 13, 1524. 34452389 10.3390/v13081524 PMC8402754

[27] Muradrasoli S (2009) Broadly targeted multiprobe qPCR for detection of coronaviruses: coronavirus is common among mallard ducks (*Anas platyrhynchos*). Journal of Virological Methods 159, 277–287. 19406168 10.1016/j.jviromet.2009.04.022 PMC7112901

[28] Joffrin L (2020) Bat coronavirus phylogeography in the Western Indian Ocean. Scientific Reports 10, 6873. 32327721 10.1038/s41598-020-63799-7 PMC7181612

[29] Tong S (2008) Sensitive and broadly reactive reverse transcription-PCR assays to detect novel paramyxoviruses. Journal of Clinical Microbiology 46, 2652–2658. 18579717 10.1128/JCM.00192-08 PMC2519498

[30] Wilkinson DA (2012) Identification of novel paramyxoviruses in insectivorous bats of the Southwest Indian Ocean. Virus Research 170, 159–163. 22982204 10.1016/j.virusres.2012.08.022

[31] Mélade J (2016) An eco-epidemiological study of Morbilli-related paramyxovirus infection in Madagascar bats reveals host-switching as the dominant macro-evolutionary mechanism. Scientific Reports 6, 23752. 27068130 10.1038/srep23752 PMC4828640

[32] Héré L (2009) Contribution à l’étude des chiroptères de l’île de La Réunion. Répartition et habitats prioritaires en matière de conservation. *Université de La Réunion, Rapport de stage de Master 2*.

[33] R Core Team (2021) R: A language and environment for statistical computing. R Foundation for Statistical Computing, Vienna, Austria. Available at https://www.R-project.org/.

[34] Bates D (2015) Fitting linear mixed-effects models using lme4. Journal of Statistical Software 67, 1–48.

[35] Fox J and Weisberg S (2019) An R Companion to Applied Regression, 3rd Edn. Los Angeles: SAGE.

[36] Baker KS (2014) Viral antibody dynamics in a chiropteran host. Journal of Animal Ecology 83, 415–428. 24111634 10.1111/1365-2656.12153 PMC4413793

[37] Hayman DTS (2018) Maternal antibody and the maintenance of a lyssavirus in populations of seasonally breeding African bats. PLoS ONE 13, e0198563. 29894488 10.1371/journal.pone.0198563 PMC5997331

[38] Montecino-Latorre D (2020) Reproduction of East-African bats may guide risk mitigation for coronavirus spillover. One Health Outlook 2, 2. 33824945 10.1186/s42522-019-0008-8 PMC7149079

[39] Baker ML, Schountz T and Wang L-F (2013) Antiviral immune responses of bats: a review: antiviral immune responses of bats. Zoonoses and Public Health 60, 104–116. 23302292 10.1111/j.1863-2378.2012.01528.x PMC7165715

[40] Han H-J (2015) Bats as reservoirs of severe emerging infectious diseases. Virus Research 205, 1–6. 25997928 10.1016/j.virusres.2015.05.006 PMC7132474

